# Missing compared to what? Revisiting heritability, genes and culture

**DOI:** 10.1098/rstb.2017.0064

**Published:** 2018-02-12

**Authors:** Marcus W. Feldman, Sohini Ramachandran

**Affiliations:** 1 Department of Biology, Stanford University, Stanford, CA 94305-5020, USA; 2 Department of Ecology and Evolutionary Biology, Brown University, Providence, RI 02912, USA

**Keywords:** kin correlations, heritability, cultural transmission, SNPs

## Abstract

Standard models for the determination of phenotypes from genes are grounded in simple assumptions that are inherent in the modern evolutionary synthesis (MES), which was developed in the 1930s, 1940s and 1950s. The MES was framed in the context of Mendelian genetic transmission enhanced by the Fisherian view of the way discretely inherited genes determine continuously quantitative phenotypes. The statistical models that are used to estimate and interpret genetic contributions to human phenotypes—including behavioural traits—are constructed within the framework of the MES. Variance analysis constitutes the main tool and is used under this framework to characterize genetic inheritance, and hence determination of phenotypes. In this essay, we show that cultural inheritance, when incorporated into models for the determination of phenotypes, can sharply reduce estimates of the genetic contribution to these phenotypes. Recognition of the importance of non-genetic transmission of many human traits is becoming ever more necessary to prevent regression to the debates of the 1970s and 1980s concerning policies based on genetic determination of complex human phenotypes.

This article is part of the theme issue ‘Bridging cultural gaps: interdisciplinary studies in human cultural evolution’.

## 1. Introduction

The explosion of genome-wide association (GWA) studies over the past 10 years might lead one to believe that scientists understand how complex human phenotypes are determined. The wording of many GWA papers suggests that the authors are unaware of the heated debates concerning the utility of the heritability statistic that occurred between 1969 and 1982. These debates, in academic and public forums, were often focused on intelligence (and by proxy measures like IQ), personality traits, or attitudes, and the extent to which these are genetically determined.

GWA studies have found thousands of DNA variants that are statistically associated with human phenotypes. The phenotypes studied are most frequently diseases, but many non-disease characteristics, such as height [1–3], children's educational achievement [4–6], economic and political preferences [7] and intelligence [8,9], have also been subjected to such DNA association analyses. In every case the amount of variance attributable to genetic differences in the measured trait is less, usually far less, than earlier estimates based on correlations between relatives. This difference is often called ‘missing heritability’ [3] and considerable effort has been expended in augmenting the heritability estimated from GWA studies to bring them closer to the higher values obtained from family studies. In what follows, we place these family studies in some historical context and ask why there should be a focus on ‘heritability’, missing or not.

In the early 1970s, at Stanford University, William Shockley, a Nobel prize–winning professor of engineering, was expounding his profoundly racist eugenic views on intelligence [10]. At the same time, Arthur Jensen, a professor of educational psychology at the University of California, was promoting similarly eugenic views that he expressed in his notorious monograph [11], which begins: ‘Compensatory education has been tried and it has apparently failed.’ Jensen blames this failure on the poor genetic endowment of those who perform badly at school^1^ [12,13].

Jensen chose IQ as the measure of likelihood to succeed in school, and focused on heritability as a measure of the extent of genetic determination. Heritability is denoted by *h*^2^, where, according to Jensen, *h* ‘tells us the correlation between genotypes and phenotypes in the population’ [11, pp. 42–43]. For IQ, Jensen suggested an average value for *h*^2^ of 0.81. However, in his assessment, the ‘most satisfactory’ [11, p. 47] and ‘most interesting’ [11, p. 52] estimate of *h*^2^ for IQ was by Burt [14], namely 0.86. Burt's studies were, however, discredited by Kamin [15] as having been based on fraudulent data, a few years after Jensen's 1969 laudation of Burt (see also Kamin [16]).

The reductionist ferment of the early 1970s was the context in which Cavalli-Sforza & Feldman produced their first model for cultural transmission and gene-culture coevolution [17]. They showed that estimates of heritability, which had been interpreted as demonstrating that such human quantitative traits as IQ were mostly genetically determined, could be obtained under vertical cultural transmission; that is, cultural transmission of the trait from parents to offspring. The model used by Cavalli-Sforza & Feldman [17] was defined by a simple dynamic recursion system in which an offspring's phenotype was determined by its genotype and its parents' phenotypes, the latter by direct vertical cultural transmission. At equilibrium of the recursions, correlations between relatives were computed as functions of all transmission parameters, and it was shown that vertical cultural transmission had a profound effect on correlations between relatives, an effect that could be misinterpreted as being due to genetic variation. In other words, cultural transmission from parent to offspring can mimic genetic heritability, and researchers should account for this vertical cultural transmission to avoid inflated estimates of *h*^2^.

## 2. Path analysis, ACE models and cultural transmission

From the 1970s to the 1990s, path analysis was widely used in the statistical analysis of complex human phenotypes, such as IQ. Sewall Wright used path analysis for estimating familial correlations under a linear model [18]. He applied path analysis to data on IQ of biological and adopted children that had been collected by Burks [19]. The linear model underlying Wright's and most subsequent path analyses of IQ is usually called ‘ACE’, where ‘A’ refers to the additive genetic contribution to a child's phenotype, ‘C’ is the contribution from environment common to or shared by offspring reared together in the natal home, and ‘E’ represents environmental contributions unique to each offspring. The phenotype of a child (e.g. IQ) is
2.1
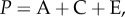
and the data consist of values of *P* for parents and offspring, either true or adopted. From parent-offspring and sib-sib correlations (and sometimes correlations between other relatives) the path coefficients, representing the ‘causes’ of *P*, are estimated.

Newton Morton's group at the University of Hawaii [20,21] applied path analysis to a collection of familial IQ correlations that included Burks' data and those published by Jencks [22], and estimated the heritability [20] of IQ to be *h*^2^ = 0.75, with a contribution *c*^2^ from shared environment of *c*^2^ = 0.09. Wright's estimate of *h*^2^, for Burks' data only, was 0.50, and was described [20] as ‘in at least qualitative agreement’ with 0.75. These estimates refer to the analysis of children's variance, but for adults (i.e. parents) the estimates are very much lower [20, table 15].

Re-analysis by Morton's group [21] of Burks' data [19] produced a slightly lower estimate of *h*^2^, namely 0.67, while the estimate of *c*^2^ remained close to 0.09. In all of these analyses [20,21], mating was assumed to be random; that is, there was no assorting for IQ.

In the late 1970s, a series of papers appeared that analysed dynamic models with genetic and cultural transmission together with assortative mating [23–26], namely the choice of mates based on phenotypic similarity. These analyses included larger sets of data and were remarkable in showing that the estimated correlation between the IQs of spouses was close to 0.5, and that the estimate of genetic heritability was much lower than all previous estimates, namely 0.32 [24] and 0.30 [26]. Even more remarkable was that the estimated fraction of variance due to cultural inheritance was not trivial relative to the heritability, namely 0.27 [25] and 0.29 [26].

There was more to come! In 1982, Morton's group made another path analysis [27] of a somewhat larger dataset of IQ correlations among American family members. This time (and without citing their earlier estimates of 0.75 and 0.67) their estimate of genetic heritability, *h*^2^, was 0.31 with 0.42 for cultural heritability, *c*^2^. Thirteen years later, Otto *et al*. [28] applied the path analysis method to sixteen familial correlations for IQ published by Bouchard & McGue [29]. The estimated heritability depended on the type of assortative mating (social homogamy or phenotypic homogamy) and whether cultural transmission was direct or indirect. The estimates of *h*^2^ varied from 0.29 to 0.42, while that of *c*^2^ was close to 0.27.

One of the reasons for the low estimates of *h*^2^ is that the correlations between the non-transmitted environments of dizygous and monozygous twins are not assumed to be equal. In addition, the correlation between the environments of monozygous twins reared apart, which had usually been ignored (set to zero), turns out not to be small [28]: even when reared apart, twins are likely to have similar environments, for example, two homes within the same extended family. On the basis of the heritability estimates between 0.30 and 0.32, Morton's group concluded [27, p. 197] ‘all analyses appear to rule out high heritability’.

## 3. Heritability and twins, again

Twelve years after the publication in leading genetics journals of 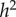 estimates close to 30%, Herrnstein & Murray write in *The Bell Curve* [30] on p. 105: ‘In fact IQ is substantially heritable … but half a century of work … permits a broad conclusion that the genetic component of IQ is unlikely to be smaller than 40% or higher than 80%. The most unambiguous direct estimates, based on identical twins reared apart, produce some of the highest estimates of heritability … we will adopt a middling estimate of 60% heritability’. Analysis of this book was public and intense: a 715-page book, *The Bell Curve Debate*, attests to the broad variety of responses the book engendered [31]. Would Herrnstein & Murray have written their 845-page tome [30] 25 years after Jensen's notorious document [11] if they had believed the heritability of IQ to be 30%?

Thirty years after the debates described above, some investigators persist in regarding heritability, computed from analyses of twins, as saying something useful about the biological aetiology of human behavioural traits [32]. Turkheimer [33, p. 26] points out that ‘unless the twin studies were somehow mistaken, covariation between DNA and behavioural differences is inevitable.’ Thus almost all human complex traits show some level of heritability as inferred from correlations between relatives. Earlier, Turkheimer [34] introduced the term ‘weak genetic explanation’ to describe this statistical phenomenon, and stresses [33, p. 24] that this weak explanation does not entail that ‘complex individual differences have genetic mechanisms for scientists to discover.’

It has been known for decades that the phenotype produced by a genotype in one environment may be radically different in another environment [35–37]. The same can be said about partitions of IQ variance in different environments. Nisbett *et al*. [38] review a number of studies of cognitive abilities in samples of families that differed on some SES-related measures [39–43]. They conclude that ‘the heritability of cognitive ability is attenuated among impoverished children and young adults in the United States.’ These findings may relate to the concept of norm of reaction. The norm of reaction is a ‘table of correspondence between phenotype, on the one hand, and genotype-environment combination on the other' [44]. In some environments, phenotypic variation among genotypes may vary a lot on the phenotype scale (high heritability), while in other environments phenotypic variation among genotypes may be small (low heritability); this is one explanation for higher heritabilities estimated from twins in higher SES environments.

The relationship between SES and heritability of cognitive ability described above points to the likelihood of cultural and/or social factors that comprise an important component of the relevant environment, which may not fit naturally into the linear analysis of variance framework that underlies heritability estimates from correlations between relatives. Evolution of aspects of the environment may result in changes in the statistics of familial relationships in cognitive ability. Dickens & Flynn [45,46] call this the ‘social multiplier’ effect, although it can be regarded as part of the evolutionary process under cultural transmission that underpinned the model of Cavalli-Sforza & Feldman [17]. Nisbett *et al*. [38] review some aspects of the environment that may correlate with SES, that may affect familial statistics of cognitive ability, and that are plausibly culturally transmitted.

Turkheimer [47] suggested that the results of decades of studies of correlations of behavioural traits between relatives to that date could be summarized by ‘Three Laws of Behaviour Genetics':

(1) All behavioural traits are heritable. (We interpret this as *h*^2^ > 0.)
(2) The effect of being raised in the same family is smaller than the effect of ‘genes’. (Our quotes added to indicate that ‘genes’ here refers to the A component in the linear variance ACE model rather than something from the DNA sequence.)
(3) A substantial portion of the variance in complex human behavioural traits is not accounted for by the effects of genes or families.

These laws apply equally well to traits such as IQ, cognitive level, years of schooling, body mass index and most other complex non-behavioural traits.

## 4. Recent meta-analyses

A remarkable meta-analysis of twin studies by Polderman *et al*. [48] appeared in 2015. They evaluated variance components for almost 18 000 traits in 2748 publications including 14 558 903 twin pairs. They report *h*^2^ = 0.49 and *c*^2^ = 0.17 ‘across all traits’, that twin resemblance is solely due to additive genetic variation, and that ‘the data are inconsistent with substantial influences from shared environment or non-additive genetic variation’. Two ways of estimating heritability were used in this massive meta-analysis. Method 1 uses 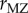 and 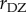, the correlations between monozygous and dizygous twins, respectively, and calculates 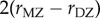 as the genetic heritability 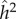, and 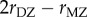 as the common environment component 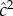 [49, p. 172]. These estimates, which are frequently used in twin studies, are to be compared with those from Method 2, namely *h*^2^ and *c*^2^, respectively, which are derived from the ACE model.

Polderman *et al*. [48] organized traits into groups (which they called ‘functional domains’) and present both heritability estimates for each group of traits. Our interest here is focused on the groups they called ‘cognitive’. For the functional domain designated ‘cognitive’, 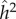 and 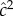 were 0.55 and 0.10, respectively, while *h*^2^ and *c*^2^ were 0.47 and 0.18, respectively. These functional domains were subdivided, and one subgroup was designated as ‘higher-level cognitive function’ for which the estimate of 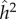 and 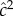 were 0.54 and 0.17, respectively. For this subgroup *h*^2^ and *c*^2^ were 0.55 and 0.18, respectively. The earlier lower estimates of family-based heritability, 30–40%, are not cited in this meta-analysis. Falconer & Mackay [49] point out that the use of 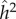 relies on the environmental components of variance in MZ and DZ twins being the same. In fact, they go on to list [49, pp. 172–173] seven ways in which a difference in these environmental components could be produced. Not included among these seven are differences in cultural transmission, either vertical or horizontal, that can affect MZ and DZ twins differently [28], for example, due to parents actively trying to treat each member of an MZ twin pair differently, or peers treating MZ twins differently from DZ twins. As has been pointed out many times [44,50,51], Polderman *et al*.'s estimate of 49% ‘across all traits’ does not, as they claim, tell us about ‘the causes of individual differences in human traits’ nor will it ‘guide future gene-mapping efforts.’ That is, heritability in general does not imply a genetic causal mechanism.

In the genomic era, intelligence has been the subject of several genome-wide association studies, the largest of which is a recent meta-analysis by Sniekers *et al*. [52]. This study included 78 308 people from 13 cohorts. Various measures of intelligence were used in eight of the cohorts, and the other five used Spearman's *g* (a statistical measure computed from a factor analysis of correlations among a number of psychometric tests of intelligence—the so-called general intelligence factor). There is remarkable heterogeneity among the measures of intelligence in the thirteen cohorts. In the two largest cohorts (54 119 individuals), intelligence was assessed by the number of correct answers out of thirteen questions produced in two minutes. The full meta-analysis included more than 12 million single nucleotide polymorphisms (SNPs). The paper begins with the announcement in the abstract that the heritability of IQ is 54%, which is actually the value of 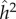 reported in the meta-analysis by Polderman *et al*. [48] and computed from 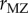 and 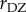. Using the SNPs, and a recent variance analysis method called ‘polygenic score regression’, Sniekers *et al*. [52] obtain an estimate of 20% for the heritability of intelligence. However, again using polygenic scores [53], the meta-analysis was only able to explain between 2% and 4.8% of the variance in four other studies, the largest of which had 9904 samples. Meta-analysis of these 9904 samples explained 2% of the phenotypic variance, while the 4.8% represented results from meta-analysis of a subset of 1558 samples. The laudatory *Nature* editorial [54] sums up these statistics by stating: ‘The associations … could explain up to 4.8% of the variance in intelligence across these cohorts.’ Even if one believed in the underlying linear statistical models that gave this result, 4.8% does not seem worth writing home about. Indeed, as Nisbett *et al*. [38, p. 135] write, ‘It may simply be that the number of genes involved in an outcome as complex as intelligence is very large, and therefore the contribution of any individual locus is just as small as the number of genes is large.’ In fact, Chabris *et al*. [55] augment Turkheimer's three laws of behaviour genetics with a fourth law that summarizes many GWAS studies of behavioural traits.

(1) A typical human behavioural trait is associated with very many genetic variants, each of which accounts for a very small percentage of behavioural variability.

## 5. Personality and attitudes

Cognitive ability assessed through IQ tests is just one of the many complex human behavioural traits whose ‘genetics’ has been investigated using data from twins. Personality traits such as extraversion and neuroticism are among those that have received most attention. Variation between twins in aspects of personality assessed using responses to questionnaires were detailed by Eaves *et al*. [56]. The dimensions of personality in this analysis were psychoticism, extraversion, neuroticism, and a ‘lie’ scale ‘designed to identify subjects responding in a ‘socially desirable’ manner’ [56, p. 74]. They concluded from these studies that there is an ‘overwhelming and consistent pattern’ of ‘a significant genetic component’ to all four personality measures with ‘no trace of a shared environmental component of twin resemblance’ [56, p. 121]. Interestingly, 24 years later one of these authors wrote ‘the structure of personality is inherent in the evolved phenotype, and is not the immediate consequence of either genetic or environmental organizing factors’ [57, p. 761].

Martin *et al*. [58] used a model similar to that of Rice [26] to analyse data on social attitudes of MZ and DZ twins, supplemented by data on social attitudes of spouses. From one set of data a composite score for conservatism was derived from dichotomous answers by Australian twins to a fifty-item questionnaire. A second dataset was used to produce composite scores for radicalism and tough-mindedness derived from a forty-item questionnaire with items on a five-point scale. Inclusion of a sample of spouses allowed estimation of the degree of assortative mating for social attitudes. For the British radicalism sample, 72% of the variation in males was found to be genetic and the cultural component zero, while in females 24% was genetic and 12% was cultural. For the Australian conservatism sample, the genetic components in males and females, respectively, were 56% and 69%, while the cultural component in both was estimated to be zero.

Martin *et al*. concluded [58] that their data on social attitudes were ‘largely consistent’ with a genetic model for family resemblance with ‘little evidence of vertical cultural transmission’. In reviewing this work on social attitudes, Eaves *et al*. [56] went further: ‘we may find that genetic differences between people are partly responsible for the distinction between godly and ungodly and between liberal and conservative in contemporary societies’ [56, p. 358]. However, a comprehensive review of such studies was made by Turkheimer *et al*. [59, p. 520], who document, in particular, the history of heritability estimates for personality traits. They summarize this history as follows: ‘One can identify broad dimensions of behaviour; quantify their relation to a broad spectrum of genes; and obtain consistent replicable results that fail to differentiate among behaviours and become uninteresting once they are established. Under most circumstances, both extraversion and introversion are heritable at approximately 0.4, and there is little more to be said.’ Again, it should be stressed that the existence of a genetic causal mechanism cannot be inferred from such statistics.

## 6. On the interpretation of heritability

The danger inherent in these studies of human behavioural, attitudinal, or personality variation resides in the meaning of ‘heritability’, whether estimated from ACE models or from statistical analysis of GWA studies. Morton [60, p. 327] makes the point succinctly: ‘one would be quite unjustified in claiming that heritability is relevant to educational strategy.’ That is, heritability estimated from familial correlations or from models designed to analyse GWA studies (although the latter began some 35 years after Morton was writing), and whether it is 5% or 95%, is not informative about the chance that environmental intervention will affect the trait under study. Despite Morton's admonition, we still see claims such as the following [61] made in 2016: ‘… soon a bit of saliva or blood from a newborn will be able to capture her full genetic potential for educational attainment …’ followed by ‘now that we have mapped the genetic architecture behind a wide range of outcomes—from height to cognitive ability—a brave new world has opened up whereby we can select our mates, and yes, even our children, by and for their genotypes’. Whereas this is plausible for relatively simple genetic traits, e.g. Mendelian diseases, it is quite implausible for height, educational attainment, cognitive ability or personality.

How are geneticists and/or social scientists to interpret estimates of heritability made from linear statistical models for familial phenotypic relationships or the contributions of genomic polymorphisms to phenotypes? We must start from recognition that all complex human traits result from a combination of causes. If these causes interact, it is impossible to assign quantitative values to the fraction of a trait due to each, just as we cannot say how much of the area of a rectangle is due, separately, to each of its two dimensions. Thus, in the analyses of complex human phenotypes, such as those described above, we cannot actually find ‘the relative importance of genes and environment in the determination of phenotype’ [44].

To illustrate their sceptical view of genetic interpretation of the heritability of personality traits, Turkheimer *et al*. [59, p. 532] consider marital status: ‘Divorce is heritable [62], but do we really expect that twin studies of marital processes will lead us to a genetic explanation of divorce? … The point is not that they are environmental as opposed to genetic; indeed as we cannot emphasize enough, marriage, divorce and whatever may cause them are just as heritable as anything else.’ But this heritability does not mean that either is ‘a biological process awaiting genetic analysis … they do not have a specific genetic aetiology.’

## 7. Culture transmission and heritability

In analyses of familial correlations for human traits, the linear statistical models that give estimates of amounts of phenotypic variance due to genes and environments (and hence heritability) rarely specify what constitutes the environment. Cavalli-Sforza & Feldman [17] took the parents' phenotypes to represent the environment in which an offspring develops its own phenotype, measured on the same scale as those of its parents, even though properties of parents other than those measured on the scale the phenotype are likely to have strong effects on that offspring's phenotype, as in the case of parental SES and children's IQ mentioned above [40]. Subsequent treatments followed Cavalli-Sforza & Feldman [17] and incorporated vertical cultural transmission (i.e. of phenotype from parent to child) into analyses of variance in IQ, attitudes and other traits [24–27,56,58].

All models that incorporate genetic and vertical cultural transmission involve a dynamic process for the evolution of the phenotype and statistical analysis of familial correlations at the stationary state. A recent analysis by Feldman *et al*. [63] extended this class of models by making specific assumptions about how the different parental pairs of phenotypes contribute probabilistically to their offspring's phenotype. The simplest such case assumes a single gene, with genotypes *AA*, *Aa*, and *aa*, and two variants of a phenotype, labelled 1 and 2. Thus there are six phenogenotypes: *AA*_1_, *AA*_2_, *Aa*_1_, *Aa*_2_, *aa*_1_, *aa*_2_. The probability that an offspring acquires phenotype 1 depends on its own genotype but not on those of its parents; it does, however, depend on their phenotypes. The general form of such phenogenotypic transmission is shown in table 1 [63].

**Table 1.** Rules of phenogenotypic transmission.

parental phenotypes M × F	offspring phenogenotype probability (given offspring’s genotype)					
	*AA*_1_	*AA*_2_	*Aa*_1_	*Aa*_2_	*aa*_1_	*aa*_2_
1 × 1	*α*_1_	1 − *α*_1_	*α*_2_	1 − *α*_2_	*α*_3_	1 − *α*_3_
1 × 2	*β*_1_	1 − *β*_1_	*β*_2_	1 − *β*_2_	*β*_3_	1 − *β*_3_
2 × 1	*γ*_1_	1 − *γ*_1_	*γ*_2_	1 − *γ*_2_	*γ*_3_	1 − *γ*_3_
2 × 2	*δ*_1_	1 − *δ*_1_	*δ*_2_	1 − *δ*_2_	*δ*_3_	1 − *δ*_3_

Although the framework exhibited in table 1 is quite simple, it does involve 12 phenogenotypic transmission rates. For this reason, Feldman *et al*. [63] simplified the transmission rule in table 1 to a form they called ‘bilinear transmission’, shown in table 2. In table 2, *β* is a baseline probability that any offspring, regardless of its genotype or parents' phenotypes, acquires phenotype 1; *α* is a transmission component due to an offspring carrying an *A* allele, with *σ* a measure of genetic dominance of *A* over *a*; *η* is the contribution to the offspring's chance of carrying phenotype 1 by each parent who carries phenotype 1, with *τ* a measure of marital dominance in transmission of phenotype 1. Thus, if 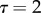, for example, a parental couple only one of whom has phenotype 1 transmits this phenotype at the same rate as a couple both of whom are of phenotype 1. The final parameter in this model is the rate *m* at which parents mate assortatively.

**Table 2.** Bilinear transmission scheme.*

mating M × F	probability that phenotype offspring is 1		
	*AA*	*Aa*	*aa*
1 × 1	*α*_1_ = 2*η* + 2*α* + *β*	*α*_2_ = 2*η* + *σα* + *β*	*α*_3_ = 2*η* + *β*
1 × 2	*β*_1_ = *γ*_1_ = *τη* + 2*α* + *β*	*β*_2_ = *γ*_2_ = *τη* + *σα* + *β*	*β*_3_ = *γ*_3_ = *τη* + *β*
2 × 1	*β*_1_ = *γ*_1_ = *τη* = 2*α* + *β*	*β*_2_ = *γ*_2_ = *τη* + *σα* + *β*	*β*_3_ = *γ*_3_ = *τη* + *β*
2 × 2	*δ*_1_ = 2*α* + *β*	*δ*_2_ = *σα* + *β*	*δ*_3_ = *β*

*See table 1 for the definition of *α_i_*, *β_i_*, *γ_i_*, *δ_i_*, 0 ≤ *σ*, *τ* ≤ 2. All transmission probabilities are nonnegative, e.g. 0 ≤ 2*η* + 2*α* + *β* ≤ 1.

Since there is no selection in the model, the frequency, *p*, of allele *A*, does not change, and the evolutionary dynamics of the six phenogenotypes can be specified in terms of the frequency *k* of phenotype 1 and the frequency of allele *A* among individuals of phenotype 1. The dynamics converge to an equilibrium at which all the usual correlations between relatives, as well as additive effects of alleles *A*_1_ and *A*_2_ [49, pp. 112–115] can be computed. The latter are used to derive the actual narrow-sense heritability, 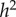, of the phenotype, namely 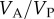, where 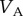 is the additive genetic variance and 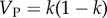 is the phenotypic variance of the population at equilibrium. From the equilibrium values of the correlations between MZ and DZ twins, we can also calculate 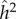 and 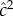, for different values of 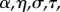 and *m*. Examples with assorting rate *m* = 0.5 are shown in table 3; *m* = 0.5 is chosen because it is very close to the value estimated for radicalism and tough-mindedness from the 562 British spousal pairs in Martin *et al*. [58].

**Table 3.** Estimates of heritability for bilinear transmission*.

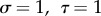			
	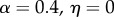	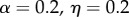	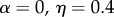
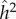	0.234	0.058	0
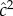	0	0.177	0.48
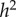	0.276	0.127	0
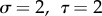			
	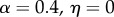	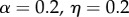	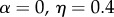
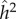	0.48	0.130	0
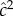	0	0.071	0.418
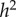	0.312	0.145	0

*Spouse correlation assumed to be 0.5 (see Martin *et al*. [58]).

The parameter sets in table 3 were chosen to represent cases of table 2 in which there is only genetic determination (*α* = 0.4, *η* = 0); genetic determination and parental transmission are equally important (*α* = 0.2, *η* = 0.2); and there is only parental transmission (*α* = 0, *η* = 0.4). As in Polderman *et al*. [48],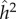 is computed as 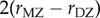 and 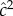 is computed as 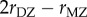 while 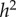 is the actual narrow sense heritability, 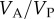. As expected, when *η* = 0 also 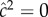, and when *α* = 0, 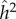 and 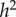 are also both zero. However, when *α* = *η* = 0.2, the dominance parameters *σ* and *τ* become important. With no dominance (*σ* = *τ* = 1) the environmental fraction of the phenotypic variance is about three times the genetic fraction, but with complete genetic and marital dominance (*σ* = *τ* = 2), the genetic value is almost twice the environmental contribution. In both cases, there are substantial discrepancies between 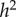 and 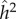, but again the direction of these differences depends on the levels of dominance.

The important feature of the model in table 2 is that it is not a linear statistical model designed for analysis of variance. It is an explicitly causative model from whose dynamic equilibrium familial and population statistics can be computed. In this simple model, the commonly computed variance estimates 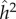 and 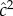 do not reflect the relative importance of genetic and environmental causation.

## 8. Concluding remarks

The biomedical and behavioural science literature over the past ten years has seen a deluge of GWA papers attempting to find common DNA markers that might be statistically associated with complex human behavioural phenotypes. As sample sizes have increased, more such markers have been found, but in few reports has the extent of environmental contribution to disease or behavioural phenotypes been taken very seriously. Epigenetic phenomena, e.g. methylation, have in some cases been shown to be influenced by such culturally transmitted environmental variables as diet or stress, but the scientific literature's focus has consistently been on common DNA polymorphisms whose effects on the phenotype under study have almost always been small [1,64,65]. Further, the contributions of these significant polymorphisms to the phenotypic variance have been small enough relative to the variance attributed to genes in analysis of familial contributions that the term ‘missing heritability’ entered the lexicon.

Missing compared to what? In the first part of this note, we discussed the limitations of estimates of heritability from familial correlations, in particular their reliance on linear models and irrelevance with respect to potential environmental interventions. Why, then, should such heritabilities be the standards relative to which GWA-based variance analyses are compared? By including those polymorphisms that failed to be significant in GWA studies, analyses of new linear models [2,66] have produced increased estimates of the variance fraction explained by genomic variation. However, it almost always remains below that estimated from familial analyses.

That heritability of a trait estimated from correlations between relatives is specific to the population in which the trait was assessed has been known for decades [50,51]. Cigarette smoking in U.S. adolescents and young adults is an example where twin-based heritability differs between whites and African-Americans [67]. A recent analysis of eight phenotypes on genomic data from the 1000 Genomes Project reference panel showed that such summary statistics as polygenic risk scores or heritability, derived from a GWAS in one population (e.g. Europeans), ‘may have limited portability to other populations’ [68]. It is also the case that genetic mutations that cause a phenotype in a population in one environment may produce an entirely different phenotype in members of a diaspora of that same population because of the changed environment experienced by the latter [69].

Recent analyses of GWAS datasets for height and schizophrenia [65] have arrived at the conclusion that the effects of SNPs that actually influence complex phenotypes are likely to be extremely small. For example, more than 100 000 SNPs ‘exert independent causal effects on height’. This extreme polygenicity, termed ‘omnigenicity’ [65], also characterizes schizophrenia, for which it is inferred that ‘broadly expressed genes contribute more to overall heritability than do brain-specific genes’. If all genes have some interaction with causal genes, it can be predicted that gene-environment interactions, even if important for causal genes, will be difficult to detect because they are likely to have small genome-wide effects whose sum may exceed the magnitude of such interactions with causal genes. Nisbett *et al*. [38, p. 135] are not optimistic: ‘This problem is not likely to be solved by advances in genetic technology that are foreseeable at present.’

The language used to interpret heritability has not changed much with advances in genomics, despite occasional genuflections towards its inability to assign causes and to gene-environment/gene-culture interactions. We still find statements on heritability such as ‘the same genes affect intelligence from age to age’ [9, p. 100], ‘intelligence shares genetic causes with education and social class’ [9, p. 104], and, referring to familial and SNP-based heritability estimates, ‘the same genes influence intelligence and social epidemiologists' ‘environmental’ variables of education, social class and height’ and ‘can enlighten research in health and social inequalities’ [9, p. 106].

Although there have been minor changes in the lexicon surrounding the calculation of heritability, due to the evolution of genomic technology, the problem of the meaning of heritability has not gone away. Heritability estimated from linear models for variance analysis still depends on the environment in which it is measured, and an increase in SNP-based heritability of cognitive performance from 10% to 30% cannot provide useful information as to whether cultural or environmental intervention is likely to have an effect. It is almost 50 years since heritability of human traits became discredited as an indicator of genetic causation. To those who were around when Jensen's monograph appeared in 1969, it must seem like déjà vu all over again.

## Endnote

1 Jensen's article appeared in the spring 1969 issue of the *Harvard Educational Review*. The summer 1969 issue of the same journal contained several critical responses to Jensen's thesis.

## Data accessibility

This article has no additional data.
